# Association of Nuclear Receptor Coactivators with Hypoxia-Inducible Factor-1*α* in the Serum of Patients with Chronic Kidney Disease

**DOI:** 10.1155/2020/1587915

**Published:** 2020-08-20

**Authors:** Tianbiao Zhou, Wenshan Lin, Shujun Lin, Zhiqing Zhong, Yuanyuan Luo, Zhijun Lin, Weiji Xie, Weitao Shen, Kai Hong

**Affiliations:** ^1^Department of Nephrology, The Second Affiliated Hospital, Shantou University Medical College, 515041 Shantou, China; ^2^Department of Clinical Laboratory, The Second Affiliated Hospital, Shantou University Medical College, 515041 Shantou, China

## Abstract

Nuclear receptor coactivators (NCOAs), consisting of coactivators and corepressors, dramatically enhance the transcriptional activity of nuclear receptors. Hypoxia-inducible factor-1*α* (HIF-1*α*) is a transcription factor that plays a major role under hypoxic conditions. This study was performed with the focus on the association of NCOAs with HIF-1*α* in the serum of chronic kidney disease (CKD) patients. Sixty patients with stage 5 CKD and 30 healthy controls from The Second Affiliated Hospital of Shantou University Medical College, between March 21, 2019, and October 30, 2019, were recruited in this prospective cohort study. We analyzed the serum levels of NCOAs (NCOA1, NCOA2, and NCOA3), HIF-1*α*, vascular endothelial growth factor (VEGF), etc. and assessed whether there was any relationship between these parameters and CKD disease. We found that circulating NCOA1 was positively associated with circulating NCOA2, NCOA3, and HIF-1*α*. A positive correlation was also observed between NCOA2 and NCOA1, NCOA3, HIF-1*α*, and VEGF. Furthermore, statistically significant correlations between NCOA3 and NCOA1, NCOA2, and HIF-1*α* were observed. The serum levels of VEGF in the CKD group were higher than those of the healthy control group. Circulating NCOA1 and circulating NCOA2 were negatively associated with procalcitonin. In conclusion, there was an association between circulating NCOA1, NCOA2, NCOA3, and circulating HIF-1*α*, and circulating VEGF was a risk factor for CKD disease. However, more studies should be performed to confirm this hypothesis.

## 1. Introduction

Nuclear receptors (NRs) are ligand sequence-specific regulated transcription factors that activate (or repress) steroid, retinoid, and thyroid hormone signals into hormone-regulated gene expression, which can regulate gene transcription via a classic, genomic mechanism [1, 2]. A growing appreciation of the complexity of NR-mediated gene transcription led to the concept of existence of coregulators (e.g., coactivators and corepressors) that modulate the NR function [1]. Successful activation of transcription by NRs requires “coregulators” of transcription, and these coregulators include coactivators that accomplish reactions required for activation of transcription and corepressors that suppress transcription [3]. NR coactivators (NCOAs), consisting of coactivators and corepressors, are recruited to DNA by NRs, potentiating NR-dependent gene transcription [1, 2], and dramatically enhancing the transcriptional activity of NRs. NCOAs influence receptor transcription through a variety of mechanisms, including histone acetyltransferase activity, methylation, phosphorylation, and chromatin remodeling [1, 4]. The NCOA's family consists of three members: NCOA1 (also known as SRC-1), NCOA2 (also known as SRC-2, TIF2, and GRIP1), and NCOA3 (also known as SRC-3, AIB1, ACTR, RAC3, p/CIP, and TRAM-1) [5–7]. In a previous study, NCOA2 (SRC-2) was detected in renal proximal tubular cells (HK-2) [8]. The kidney is an organ comprising of various NRs, and there has been no study regarding the role of NCOAs in renal disease.

Hypoxia-inducible factor-1*α* (HIF-1*α*) is a transcription factor that plays a major role under hypoxic conditions. Various studies reported that the increased expression of HIF-1*α* was associated with cancer risk and cancer prognosis [9–12]. However, currently, the effects of HIF and vascular endothelial growth factor (VEGF) are not well known. Some studies on renal diseases found that HIF-1*α* played a protective role [13–15], but others reported that it was a risk factor [16–18]. Current evidence indicated that HIF-1*α* could be regulated by some NR [19–21]; however, there was no report showing the relationship between NCOAs and HIF-1*α*. The VEGF is a target gene of HIF-1*α* [22].

Chronic kidney disease (CKD) is defined when the glomerular filtration rate is less than 60 mL/min/1.73 m^2^ for more than 3 months [23]. It is caused by dysfunctional kidneys and has a complex pathogenesis. The molecular mechanism of kidney disease is extremely complex, and many factors, such as NCOAs, HIF-1*α*, and VEGF, may be involved in its pathogenesis and progression. In this study, we studied the role of circulating NCOAs, HIF-1*α*, and VEGF and the relationship between them in patients with stage 5 CKD.

## 2. Materials and Methods

### 2.1. Study Population

Sixty patients with stage 5 CKD and 30 healthy controls from The Second Affiliated Hospital of Shantou University Medical College, between March 21, 2019, and October 30, 2019, were included in this prospective cohort study. Stage 5 CKD was defined as an estimated glomerular filtration rate (eGFR) less than 15 mL/min/1.73m^2^ [24]. The equation derived from the Chronic Kidney Disease Epidemiology Collaboration was used to calculate the eGFR [25]. The stage 5 CKD patients were divided into those not on dialysis (nondialysis group), those on hemodialysis (hemodialysis group), and those on peritoneal dialysis (peritoneal dialysis group). Stage 5 CKD patients with stable vital functions, who had been treated with haemodialysis or peritoneal dialysis for more than 3 months, were included in this study. The healthy control group comprised of people who visited our hospital's medical examination center for a health examination. All works in this study were conducted in accordance with the Declaration of Helsinki (1964), and the study was approved by the ethics committee of The Second Affiliated Hospital of Shantou University Medical College (SUMC2H: 2019-1), and written informed consent was obtained from all the included participants.

### 2.2. Measurement of Parameters

The clinical testing parameters were identified and extracted prospectively. The serum NCOA1, NCOA2, NCOA3, HIF-1*α*, and VEGF levels were detected by the enzyme-linked immunosorbent assay method, and all the reagents were purchased from Abcam, Co., USA (HIF-1*α*: ab171577; VEFG: ab222510) and Dogesce (NCOA1: DG95699Q; NCOA2: DG95703Q; and NCOA3: DG95709Q). The absorbance of the samples of CKD patients and healthy controls was determined at 450 nm by an enzyme-labeling measuring instrument (Fermi automatic enzyme-linked immunosorber, Switzerland). The creatinine, blood urea nitrogen, white blood cells, red blood cells, hemoglobin, platelet (PLT), albumin, magnesium (Mg), procalcitonin (PCT), calcium (Ca), phosphorus, immunoreactive parathyroid hormone, B-type natriuretic peptide, high-sensitivity C reactive protein, and creatine kinase isoenzyme MB (CK-MB) values were also determined.

### 2.3. Statistical Analysis

This study used Statistical Package for the Social Sciences version 25.0 statistical software for statistical analysis. The measurement data were compared among the three subgroups of CKD patients. If the data were normally distributed, analysis of variance was used, and the data were expressed as the mean ± standard deviation. If the data were not distributed normally, then the Kruskal-Wallis test was used and the results were expressed in quartiles [P50 (P25, P75)]. Correlation analysis was used to analyze the correlation between NCOA1, NCOA2, NCOA3, HIF-1*α*, VEGF, and other indicators, while regression analysis was used to analyze the influencing factors of NCOA1, NCOA2, NCOA3, HIF-1*α*, and VEGF. The receiver operating characteristic (ROC) curve was used to analyze the indicators of NCOA1, NCOA2, NCOA3, HIF-1*α*, and VEGF status. The significance level for all tests was set at *P* < 0.05.

## 3. Results

### 3.1. Baseline Clinical Data

This study enrolled 60 stage 5 CKD patients and 30 healthy controls. The sex distribution of male/female in the CKD group and healthy control group was 48.33% and 50%, respectively. The mean age of the participants in the CKD and healthy control groups was 53.54 ± 16.26 years and 51.18 ± 13.53 years, respectively. The leading cause of CKD was glomerulonephritis (19/60, 31.67%), followed by diabetic nephropathy (16/60, 26.67%), hypertensive nephropathy (11/60, 18.33%), obstructive nephropathy (6/60, 10%), lupus nephritis (4/60, 6.67%), immunoglobulin A nephropathy (2/60, 3.33%), and light chain deposition-associated nephropathy (1/60, 1.67%). The CKD patient distribution according to (hemodialysis/peritoneal dialysis/nondialysis) status was 20/20/20.

### 3.2. Comparison of NCOA1, NCOA2, NCOA3, HIF-1*α*, and VEGF Levels between the CKD Group and the Healthy Control Group

The VEGF level in the CKD group was notably higher than that in the healthy control group (*P* < 0.05; Table 1). Furthermore, in the subgroup analysis of the CKD group, we found that the serum levels of VEGF in the patients of the nondialysis, hemodialysis, and peritoneal dialysis groups were notably higher than those in the healthy control group (*P* < 0.05; Table 1). However, no differences in the serum levels of NCOA1, NCOA2, NCOA3, and HIF-1*α* between the CKD group and the healthy control group were found. Even in the subgroup analysis, we did not find any difference in the above parameters between the healthy control group and the nondialysis, hemodialysis, and peritoneal dialysis groups (all *P* > 0.05; Table 1).

### 3.3. Subgroup Analysis

#### 3.3.1. Correlation Analysis between Circulating NCOA1 and Other Indicators in CKD Patients

According to the correlation analysis, Table 2 shows that circulating HIF-1*α* was positively associated with circulating NCOA1. There was a significant positive correlation between circulating NCOA2 and circulating NCOA1 and a notable positive correlation between circulating NCOA3 and circulating NCOA1 (Table 2). Ca was positively associated with circulating NCOA1 (Table 2). There was a negative correlation between PCT and circulating NCOA1; however, there were no positive correlations between circulating NCOA1 and other indicators (Table 2).

#### 3.3.2. Correlation Analysis between Circulating NCOA2 and Other Indicators in CKD Patients

According to the correlation analysis, Table 3 shows that age was positively associated with circulating NCOA2. A positive association was observed between VEGF and NCOA2 in the serum, and circulating HIF-1*α* was notably associated with circulating NCOA2 (Table 3). There was a significant positive correlation between NCOA1 and NCOA2, as well as between NCOA3 and NCOA2 in the serum (Table 3). PLT was positively associated with NCOA2, but there was a negative correlation between PCT and NCOA2 in the serum (Table 3). CKMB was positively associated with NCOA2, but there were no positive correlations between NCOA2 and other indicators in the serum (Table 3).

#### 3.3.3. Correlation Analysis between Circulating NCOA3 and Other Indicators in CKD Patients

According to the correlation analysis, Table 4 shows that HIF-1*α* was positively associated with NCOA3 in the serum. A significant positive correlation between NCOA1 and NCOA3 was found, along with a notable positive correlation between NCOA2 and NCOA3 in the serum (Table 4). However, there were no positive correlations between NCOA3 and other indicators in the serum (Table 4).

#### 3.3.4. Correlation Analysis between HIF-1*α* and Other Indicators in the Serum of CKD Patients

According to the correlation analysis, Table 5 shows that NCOA1 was positively associated with HIF-1*α* in the serum. A significant positive correlation was found between NCOA2 and HIF-1*α* and between NCOA3 and HIF-1*α* in the serum (Table 5). However, there were no positive correlations between serum HIF-1*α* and other indicators (Table 5).

#### 3.3.5. Correlation Analysis between VEGF and Other Indicators in the Serum of CKD Patients

According to the correlation analysis, it can be seen from Table 6 that age was positively associated with VEGF in the serum. There was a significant positive correlation between NCOA2 and VEGF, and a marked positive correlation was observed between PLT and VEGF in the serum (Table 6). Mg was positively associated with VEGF, and there was a notable positive correlation between PCT and VEGF in the serum (Table 6). However, there were no positive correlations between VEGF and other indicators in serum (Table 6).

### 3.4. ROC Analysis for CKD Disease

**Figure 1:**
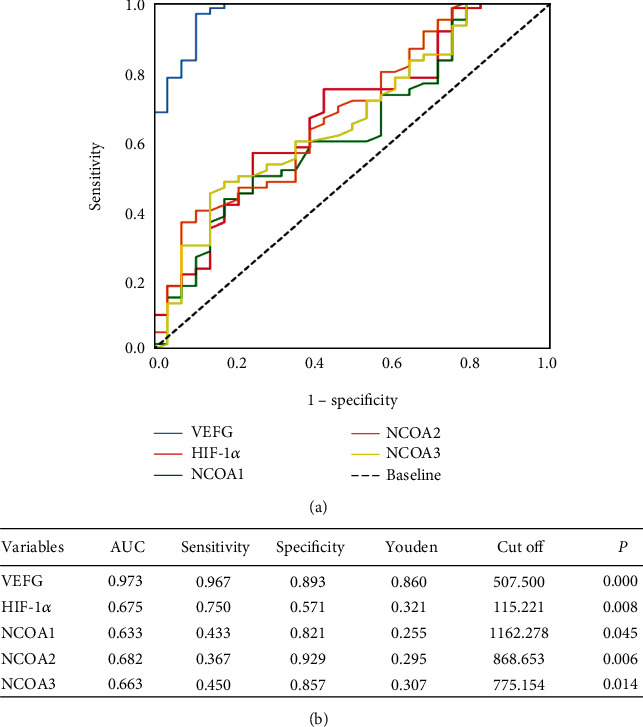
ROC curve of indicators for CKD and predictive value of indicators. (a) ROC curve of indicators for CKD. (b) ROC curve analysis of predictive value of indicators for CKD. Note: according to the ROC curve analysis, it can be seen from the above table that the AUC is closer to 1 when the AUC > 0.5, which indicates that the diagnosis effect is better. AUC has a low accuracy at 0.5 to 0.7, AUC has a certain accuracy at 0.7 to 0.9, AUC has a high accuracy at above 0.9, and AUC = 0.5, indicating that the diagnostic method is completely ineffective and has no diagnostic value.

In this ROC curve analysis, the area under curve (AUC) for the VEGF curve was represented as 0.973, at *P* = 0.000 (<0.05), which indicates that the ROC was statistically significant and the results had high accuracy. The sensitivity was 0.967, and the specificity was 0.893. Thus, the ability to identify patients correctly as ‘true positive and false positive was 96.7% and 89.3%, respectively (Figure 1).

The AUC for the serum HIF-1*α* curve was 0.675, at *P* = 0.008 (<0.05), indicating that the ROC was statistically significant and the results had lower accuracy. The sensitivity and specificity were 0.750 and 0.571, respectively, thus indicating that the ability to identify the true positives and false positives was 75.0% and 57.1%, respectively (Figure 1).

The AUC for the serum NCOA1 curve was 0.633 at *P* = 0.045 (<0.05), indicating that the ROC was statistically significant and the results had lower accuracy. The sensitivity and specificity was 0.433 and 0.821, respectively. This indicates that the ability to correctly identify the true positive and false positive was 43.3%, and 82.1%, respectively (Figure 1).

The AUC for the serum NCOA2 curve was 0.682 at *P* = 0.006 (<0.05), indicating that the ROC was statistically significant and the results had lower accuracy. The sensitivity was 0.367 and the specificity was 0.929, indicating that the ability to correctly identify true positive and false positive was 36.7% and 92.9%, respectively (Figure 1).

The AUC for serum NCOA3 was 0.663 at *P* = 0.014 (<0.05), indicating that the ROC was statistically significant and the results had lower accuracy. The sensitivity and specificity was 0.450 and 0.857, respectively. This indicated that the ability to correctly identify the true positive and false positive was 45.0% and 85.7%, respectively (Figure 1).

## 4. Discussion

In this study, we found positive correlations between NCOA1, NCOA2, NCOA3, and HIF-1*α* in the serum. Additionally, NCOA2 also showed a positive correlation with VEGF in the serum. The results indicated that there might be a signal pathway of NCOAs (NCOA1, NCOA2, and NCOA3) HIF-1*α* in the serum of CKD disease. NCOAs play crucial roles in the regulation of downstream gene expression. No other study until now has explored the expression or role of NCOAs in renal diseases. Our study is the first to reveal that in CKD disease, the NCOA1, NCOA2, and NCOA3 were associated with HIF-1*α* in the serum. The HIF-1*α* has been reportedly associated with anemia, as well as with renal interstitial fibrosis and glomerulosclerosis. However, the role of HIF-1*α* in the renal fibrosis and extracellular matrix (ECM) accumulation has been conflicting. Ke et al. [26] demonstrated that mitochondrial uncoupling protein-2 regulates renal tubulointerstitial fibrosis by stimulating the accumulation of lipid deposition and ECM, and that HIF-1*α* inhibition by siRNA can suppress the accumulation of lipid and ECM, as well as suppression of collagen I and fibronectin expression in proximal renal tubular cells. Li et al. [27] used the method of weighted gene coexpression network analysis to link the renal tubulointerstitial gene expression profile of diabetic nephropathy to eGFR values and found that there was a negative correlation between HIF-1*α* and eGFR, as well as samples with increased HIF-1*α* expression were enriched with the signaling pathways of renal fibrosis. However, Fang et al. [28] reported that knockdown of HIF-1*α* or HIF-2*α* can inhibit the miR-29c expression in renal interstitial fibrosis in humans and rats and the activation of HIF-*α* can attenuate the renal fibrosis. However, in our study, we found that the difference in HIF-1*α* between CKD patients and healthy controls was not notable. More studies should be conducted to assess the detailed role of HIF-1*α* in renal disease.

However, we found that the serum levels of VEGF in the CKD group were higher than those of the healthy control group, and this indicated that VEGF could be a risk factor for CKD disease. We also found that the serum levels of VEGF in the nondialysis group, hemodialysis group, and peritoneal dialysis group were higher than those in the healthy control group. Deletion of VEGF in podocytes destroyed the maintenance of renal vascular integrity; on the other hand, increased levels of VEGF in podocytes can cause the glomerular basement membrane thickening and mesangial expansion [29]. Zuo et al. [30] reported that VEGF is the risk factor for the diabetic CKD. Anderson et al. [31] reported that plasma VEGF level increased in patients with CKD. However, Pfister et al. [32] reported that anti-VEGF therapy induced segmental glomerular capillary microaneurysms and segmental hyalinosis. Furthermore, Erturk et al. [33] included end-stage renal disease patients and healthy participants in their study and reported that the VEGF levels in the end-stage renal disease group were not significantly higher than those in the healthy control group. The result of our study was similar to that of the studies by Zou et al. [30] and Anderson et al. [31]. Interestingly, our study was the first to report that VEGF was positively associated with NCOA2 in CKD patients. We speculated that NCOA2, as a nuclear regulatory factor, could induce the VEGF expression, which can induce renal interstitial fibrosis and glomerulosclerosis. However, further studies should be conducted to investigate this relationship.

In previous reports, it was found that HIF-1*α* was an upstream signal factor for VEGF, and HIF-1*α* could induce the VEGF expression [22, 34, 35]. However, in this clinical study, we did not find this relationship. We speculated that the underlying diseases in CKD patients might affect these results, and a positive relationship could not be found. Future studies with larger sample sizes should be conducted to study this association.

In this study, there were some limitations. First, the sample size of this study was small; hence, subgroup analysis based on the causes of CKD could not be performed. Second, sources of NCOAs in blood of CKD patients remained unclear, but it is necessary to clarify any significance of NCOA expression in renal tissues. In this study, the renal biopsy was not performed because it was considered a nonessential inspection in stage 5 CKD patients. Furthermore, associations between HIF-1*α* and NCOAs in the kidney could not be effectively determined because renal biopsy was not performed. Using animal models with CKD could be a good choice to assess this relationship.

This study was also the first to report that NCOA1 and NCOA2 were negatively associated with PCT in the serum (a validation factor predicts bacterial infection). This probably shows that NCOA1 and NCOA2 might be protective factors against bacterial infection. However, there was rare evidence regarding this, and further research should be conducted to explore it.

In conclusion, we established an association between NCOA1, NCOA2, NCOA3, and HIF-1*α* in the serum, and the circulating VEGF in CKD patients was higher than that in healthy controls. However, more studies may be needed in the future.

## Figures and Tables

**Table 1:** NCOA1, NCOA2, NCOA3, HIF-1*α*, and VEFG levels between the CKD group and healthy control group.

Variables	Nondialysis group	Hemodialysis group	Peritoneal dialysis group	Chronic kidney disease group	Healthy control group	*Z*	*P*
VEFG	727.45 (577.63, 945.40)	1047.50 (896.55, 1154.20)	1101.80 (886.95, 1146.20)	982.10 (786.03, 1140.25)	257.90 (191.53, 321.60)^abcd^	69.605	0.000
HIF-1*α*	125.10 (116.15, 153.46)	121.33 (107.44, 127.25)	127.72 (107.00, 138.43)	124.40 (109.64, 132.60)	114.29 (73.42, 126.97)	8.483	0.075
NCOA1	1220.65 (930.84, 1377.90)	1139.46 (963.10, 1649.38)	1081.63 (974.25, 1188.29)	1120.84 (974.25, 1272.05)	1048.76 (804.52, 1153.97)	6.900	0.141
NCOA2	715.08 (595.14, 876.94)	802.82 (645.33, 1002.11)	832.60 (725.42, 937.41)	772.87 (655.58, 937.41)	697.39 (448.00, 829.62)	8.793	0.066
NCOA3	750.81 (613.14, 860.40)	761.21 (683.74, 860.40)	755.41 (685.46, 827.03)	758.89 (660.15, 855.13)	717.20 (518.15, 761.21)	6.095	0.192

Compared with the nondialysis group, ^a^*P* < 0.05; compared with the hemodialysis group, ^b^*P* < 0.05; compared with the peritoneal dialysis group, ^c^*P* < 0.05; compared with the chronic kidney disease group, ^d^*P* < 0.05.

**Table 2:** Correlation analysis between NCOA1 and other indicators in CKD patients.

Variables	*r*	*P*	Variables	*r*	*P*
Age	0.177	0.099	HB	0.038	0.772
VEFG	0.061	0.573	ALB	-0.159	0.230
HIF-1*α*	0.666	0.000	Ca	-0.352	0.007
NCOA2	0.796	0.000	P	-0.026	0.859
NCOA3	0.743	0.000	Mg	-0.109	0.551
Cr	-0.052	0.695	PCT	-0.487	0.016
BUN	0.168	0.201	PTH	0.011	0.942
WBC	0.179	0.171	BNP	-0.210	0.324
RBC	0.028	0.832	hsCRP	0.234	0.123
PLT	0.193	0.139	CKMB	0.223	0.191

**Table 3:** Correlation analysis between NCOA2 and other indicators in CKD patients.

Variables	*r*	*P*	Variables	*r*	*P*
Age	0.220	0.040	HB	0.103	0.432
VEFG	0.215	0.044	ALB	-0.086	0.515
HIF-1*α*	0.701	0.000	Ca	-0.096	0.478
NCOA1	0.796	0.000	P	0.087	0.544
NCOA3	0.769	0.000	Mg	-0.146	0.426
Cr	0.093	0.480	PCT	-0.440	0.032
BUN	0.118	0.370	PTH	0.064	0.660
WBC	0.087	0.509	BNP	-0.255	0.229
RBC	0.137	0.296	hsCRP	0.046	0.762
PLT	0.323	0.012	CKMB	0.391	0.018

**Table 4:** Correlation analysis between NCOA3 and other indicators in CKD patients.

Variables	*r*	*P*	Variables	*r*	*P*
Age	0.145	0.178	HB	0.061	0.641
VEFG	0.161	0.134	ALB	-0.077	0.563
HIF-1*α*	0.684	0.000	Ca	-0.075	0.578
NCOA1	0.743	0.000	P	0.016	0.912
NCOA2	0.769	0.000	Mg	-0.004	0.984
Cr	0.084	0.525	PCT	-0.347	0.097
BUN	0.135	0.304	PTH	0.032	0.829
WBC	0.169	0.196	BNP	-0.330	0.115
RBC	0.042	0.749	hsCRP	0.036	0.816
PLT	0.166	0.206	CKMB	0.180	0.292

**Table 5:** Correlation analysis between HIF-1*α* and other indicators in CKD patients.

Variables	*r*	*P*	Variables	*r*	*P*
Age	0.157	0.143	HB	0.123	0.350
VEFG	0.143	0.185	ALB	-0.098	0.459
NCOA1	0.666	0.000	Ca	-0.158	0.240
NCOA2	0.701	0.000	P	0.149	0.298
NCOA3	0.684	0.000	Mg	0.009	0.961
Cr	0.088	0.504	PCT	-0.445	0.029
BUN	0.190	0.145	PTH	-0.045	0.759
WBC	0.045	0.734	BNP	-0.142	0.508
RBC	0.140	0.287	hsCRP	-0.001	0.997
PLT	0.055	0.674	CKMB	0.310	0.066

**Table 6:** Correlation analysis between VEGF and other indicators in CKD patients.

Variables	*r*	*P*	Variables	*r*	*P*
Age	0.213	0.046	HB	0.186	0.155
HIF-1*α*	0.143	0.185	ALB	0.036	0.788
NCOA1	0.061	0.573	Ca	0.208	0.121
NCOA2	0.215	0.044	P	0.121	0.397
NCOA3	0.161	0.134	Mg	0.382	0.031
Cr	0.115	0.384	PCT	0.456	0.025
BUN	-0.042	0.749	PTH	0.125	0.393
WBC	0.131	0.318	BNP	0.337	0.107
RBC	0.251	0.053	hsCRP	0.284	0.058
PLT	0.265	0.041	CKMB	-0.121	0.483

## Data Availability

All data generated of this study are included in the published article. The data used in this study are available on request from the corresponding author.

## Abbreviations

CKD: Chronic kidney disease
NCOA1: Nuclear receptor coactivator 1
NCOA2: Nuclear receptor coactivator 2
NCOA3: Nuclear receptor coactivator 3
HIF-1*α*: Hypoxia-inducible factor-1*α*
VEFG: Vascular endothelial growth factor
Cr: Creatinine
BUN: Blood urea nitrogen
WBC: White blood cells
RBC: Red blood cells
Hb: Haemoglobin
PLT: Platelet
Alb: Albumin
Mg: Magnesium
PCT: Procalcitonin
Ca: Calcium
P: Phosphorus
PTH: Immunoreactive parathyroid
BNP: B-type natriuretic peptide
hsCRP: High-sensitivity C reactive protein
CK-MB: Creatine kinase isoenzyme MB.
